# Destabilizing Different Strengths of Fear Memories Requires Different Degrees of Prediction Error During Retrieval

**DOI:** 10.3389/fnbeh.2020.598924

**Published:** 2021-01-08

**Authors:** Wei Chen, Junjiao Li, Liang Xu, Shaochen Zhao, Min Fan, Xifu Zheng

**Affiliations:** ^1^Key Laboratory of Brain, Cognition and Education Sciences (South China Normal University), Ministry of Education, Guangzhou, China; ^2^School of Psychology, Center for Studies of Psychological Application, Guangdong Key Laboratory of Mental Health and Cognitive Science, South China Normal University, Guangzhou, China; ^3^College of Teacher Education, Guangdong University of Education, Guangzhou, China; ^4^Guangdong Communication Polytechnic, Guangzhou, China; ^5^China People's Police University, Guangzhou, China

**Keywords:** fear conditioning, memory destabilization, reconsolidation, prediction error, ambiguity

## Abstract

Reactivation of consolidated memories can induce a labile period, in which these reactivated memories might be susceptible to change and need reconsolidation. Prediction error (PE) has been recognized as a necessary boundary condition for memory destabilization. Moreover, memory strength is also widely accepted as an essential boundary condition to destabilize fear memory. This study investigated whether different strengths of conditioned fear memories require different degrees of PE during memory reactivation in order for the memories to become destabilized. Here, we assessed the fear-potentiated startle and skin conductance response, using the post-retrieval extinction procedure. A violation of expectancy (PE) was induced during retrieval to reactivate enhanced (unpredictable-shock) or ordinary (predictable-shock) fear memories that were established the day before. Results showed that a PE retrieval before extinction can prevent the return of predictable-shock fear memory but cannot prevent the return of unpredictable-shock fear memory, indicating that a single PE is insufficient to destabilize enhanced fear memory. Therefore, we further investigated whether increasing the degree of PE could destabilize enhanced fear memory using different retrieval strategies (multiple PE retrieval and unreinforced CS retrieval). We found that spontaneous recovery of enhanced fear memory was prevented in both retrieval strategies, but reinstatement was only prevented in the multiple PE retrieval group, suggesting that a larger amount of PE is needed to destabilize enhanced fear memory. The findings suggest that behavioral updating during destabilization requires PE, and the degree of PE needed to induce memory destabilization during memory retrieval depends on the strength of fear memory. The study indicates that memory reconsolidation inference can be used to destabilize stronger memories, and the findings shed lights on the treatment of posttraumatic stress disorders and anxiety disorders.

## Introduction

Memories with extreme emotional connotations serve a pathogenic role in diverse psychiatric disorders, such as posttraumatic stress disorder (PTSD), as well as phobias (Beckers and Kindt, 2017; Chen et al., 2020). Provoking fundamental changes in the emotional impact of the memory is difficult to do in the clinical setting. However, mounting research evidence opines that memories are not set in stone even after consolidation (Misanin et al., 1968). After retrieval, consolidated memory enters a transient labile period, in which it might be vulnerable to amnestic interventions and require de novo protein synthesis for re-stabilization to persist. During this window period, memory can be modified by protein synthesis inhibitors (e.g., anisomycin) or beta receptor antagonists (e.g., propranolol) (Nader et al., 2000; Debiec and Ledoux, 2004; Kindt et al., 2009). In addition, reactivated memories are susceptible to behavioral manipulations, such as the retrieval-extinction approach (Monfils et al., 2009; Schiller et al., 2010). In the retrieval-extinction procedure, an isolated retrieval trial is presented to destabilize consolidated memory. This is followed by introduction of extinction training for updating the memory in the reconsolidation period in place of generating a second memory trace, which competes for expression. Monfils et al. (2009) first demonstrated the efficacy of this retrieval-extinction procedure when a single unreinforced conditioned stimulus (CS) given to rats shortly before extinction lessened the spontaneous recovery, reinstatement, and renewal of fear learning. Thereafter, Schiller et al. (2010) advanced these results in a human fear-training research. This experimental paradigm and its variants have been reported to extinguish fear- and addiction-related memory in humans (Bjorkstrand et al., 2016; Agren et al., 2017; Lee et al., 2017; Thompson and Lipp, 2017; Chen et al., 2019; Kitamura et al., 2020). However, reproducibility issues in the field (Kredlow et al., 2018; Zimmermann and Bach, 2020) have suggested that this effect is sensitive to procedural differences. A meta-analysis established that retrieval extinction had a negligible to moderate influence on impeding recovery of the fear responses compared with standard extinction (Kredlow et al., 2016). More work is required before the approach is applied in clinical interventions.

Memory reconsolidation is a process that incorporates new information into memories, thereby maintaining the relevance of memory traces under changing environmental conditions. Its function is to update memories in strength and content (Lee, 2009; Fernandez et al., 2016a). If no information that deviates from the memory being retrieved is present, memory reconsolidation is unnecessary and yields a likelihood for memory impediment without yielding any benefits. Accumulating evidence indicates that prediction error (PE), a discord between the anticipated as per the previous encounters and the actual condition (Rescorla and Wagner, 1972), is a pivotal condition upon retrieval to destabilize memory, as demonstrated in a series of animal and human studies (Sevenster et al., 2012, 2013; Diaz-Mataix et al., 2013; Alfei et al., 2015; Ferrer Monti et al., 2017). One of our previous functional MRI studies, which demonstrated that PE retrieval diminished inferior temporal cortex (IT), as well as dorsolateral prefrontal cortex (dlPFC) activity and IT-dlPFC and dlPFC-anterior cingulate cortex (ACC) functional connectivity during subsequent extinction training, also corroborates this perspective (Junjiao et al., 2019). During a reactivation session, various PE manipulations, including CS or unconditional stimulus (US) only, CS-US pairing, US timing differences, and change in reward contingency, may trigger memory destabilization (Fernandez et al., 2016b). However, few studies have parametrically measured the prediction error (Sinclair and Barense, 2019). Defining these factors is critical for comprehending the outcomes of prediction error.

Accumulating evidence suggests that memory destabilization occurs only after a narrow degree of PE upon memory retrieval, whereas insufficient PE leaves the memory trace in an inactive state. Importantly, an excessive degree of PE during retrieval results in extinction, during which subsequent interventions will disturb consolidation of the extinction memory trace in place of the initial fear memory trace (Sevenster et al., 2014; Alfei et al., 2015). Specifically, the degree of PE during retrieval delimits the shift from solo retrieval to reconsolidation, limbo (an intermediate state that is insensitive to amnestic agents), or extinction (Merlo et al., 2014; Faliagkas et al., 2018). Notably, more strongly encoded memories are resistant to disruption following reactivation (Visser et al., 2018; Zuccolo and Hunziker, 2019). Memory strength is widely accepted as an essential boundary condition to destabilize fear memory, and extra manipulation is needed to let the strong memory render destabilization, such as a prolonged reactivation period or some novel information during retrieval (Suzuki et al., 2004; Winters et al., 2009; Hu et al., 2018). However, whether the degree of PE needed to induce memory destabilization during retrieval is based on the strength of the memory remains unclear.

Recently, Amadi et al. (2017) found that the temporal ambiguity of the aversive events enhances fear. Since the information about the time and occurrence of aversive events is obtained quickly, unexpectedly timed or omitted aversive events generate hippocampal signals to enhance fear learning. For the present work, we modified the paradigm by Amadi et al. (2017) to form an enhanced fear memory strength with unpredictably timed shocks. On the following day, different degrees of PE were presented during reactivation sessions to retrieve enhanced or ordinary fear memories, followed by extinction training 10 min later. The spontaneous recovery and reinstatement of conditioned fear was assessed on the third day. Using this protocol, we hypothesized that PE is required in the induction of memory destabilization in retrieval extinction (experiment 1), and that the degrees of PE needed to trigger memory destabilization should increase if the strength of fear memory is enhanced (experiment 2). We tested these hypotheses in a retrieval-extinction procedure of fear instructing in humans using the fear-potentiated startle (FPS), as well as skin conductance response (SCR).

## Materials and Methods

### Participants

Eighty-nine healthy students (27 males and 62 females) from South China Normal University were enrolled in the study (mean age, 20.3 ± 2.1 years; ranging between 18 and 30 years). All study subjects were right-handed, with no history of physical or mental disorders, normal vision or corrected visual acuity, normal hearing, no recent nasal congestion or cough, and had not participated in similar studies before. A score ≥18 on the Beck Depression Inventory (BDI) (Beck et al., 1961; Bos et al., 2014) constituted an additional exclusion criterion. All the subjects provided written informed consent as per the Declaration of Helsinki and were informed that they could exit the study at any time. The Research Ethics Review Board of South China Normal University approved the study (approval number: 182). Subjects completing the study were paid (RMB 60) at the end of the study.

Subjects were randomly assigned to different groups based on matched sex, age, as well as Trait Anxiety (STAI-T) (Spielberger et al., 1970) (see Supplementary Table S1). Two subjects were excluded because of high BDI scores. In experiment 1, there were 18 subjects in the predictable-shock/PE-retrieval group (five males), 19 in the predictable-shock/no PE-retrieval group (five males), and 16 in the unpredictable-shock/PE-retrieval group (five males). In experiment 2, there were 17 subjects in the multiple PE retrieval group (five males) and 17 in the unreinforced CS retrieval group (six males).

### Stimuli

Single-color three-dimensional geometric pictures were used as CSs in experiments 1 and 2 (yellow cylinder and red cube, respectively; Supplementary Figure S1). The slides had the same brightness with a white background and were projected at the center of a 16-in. black computer screen. The presentation of one of the slides (CS+) was succeeded by two US, while that of the other slides (CS–) was not. Slide assignment (CS+ or CS–) was counterbalanced across the subjects, and both CS+ and CS– stimuli were displayed for 6 s. The startle probe defined as the 40-ms duration noise burst (104 dB sound pressure level) of a rise/fall time of <1 ms, was conveyed binaurally via earpieces. The US constituted an electrical stimulation with 200 ms and a current of 50 pulses/s, individually established at the start of the test as “uncomfortable but not painful.” “Mild” electric shocks were conveyed *via* a stimulating bar electrode connected to the right hand's wrist. Electric shocks were generated by a Digitimer DS2A-Mk.II Constant Voltage Stimulator (Hertfordshire, UK).

### Fear-Potentiated Startle

The conditioned fear response (CR) constituted the eyeblink startle reflex potentiation to a loud noise through electromyography (EMG) of the right orbicularis oculi muscle. We administered the loud noise (40 ms; 104 dB) in the deliverance of each CS, as well as intertrial intervals [noise alone (NA)]. We conveyed all of the acoustic stimuli binaurally through earpieces (HD 600, Sennheiser, Germany). FPS was measured using Xeye Human Startle Reflex (Beijing Tianming Hongyuan Technology Development Co. Ltd., China). Two 4-mm Ag/AgCl electrodes full of the electrolyte gel were positioned about 1 cm beneath the pupil, as well as 1 cm under the lateral canthus, and a ground reference was positioned on the forehead (Blumenthal et al., 2005). The EMG electrodes were linked to an evoked potential amplifier set at an input resistance of 10 MΩ, as well as a bandwidth of DC-1500 Hz. To eliminate undesirable interferences, the notch filter was set at 50 Hz. The amplitude from the beginning to the peak (over the period of 50–300 ms after the probe onset) was assessed as the startle eye-blink magnitude (microvolts) and standardized for each subject via the T-normalization, yielding a distribution with a total mean of 50, along with a standard deviation of 10 for each stage. Startle response outliers were elucidated for each day separately (*Z* > 3) and were replaced by linear trend at points (Sevenster et al., 2012, 2013).

### Skin Conductance Response

SCR was assessed by a Spirit NeXus-10 (BioTrace Medical, San Carlos, CA, USA). Signal sampling was performed at 120 Hz. The first, as well as the second phalanges of the first and second fingers of the left hand were connected using two 4-mm Ag/AgCl electrodes. We analyzed the SCR waveforms offline *via* the BioTrace+ software for NeXus-10. The SCR excited by the CS was estimated by computing the difference between the average baseline (i.e., 1 s prior to CS onset) and peak waveforms within the 1–5-s period after the onset of the stimulus. A minimum response criterion set at 0.02 micro-Siemens (μS) was utilized. We scored all the other responses as zero and were kept in the assessments. Square root transformation of the raw SCR scores was performed to standardize the distributions.

### Experimental Procedure

**Figure 1:**
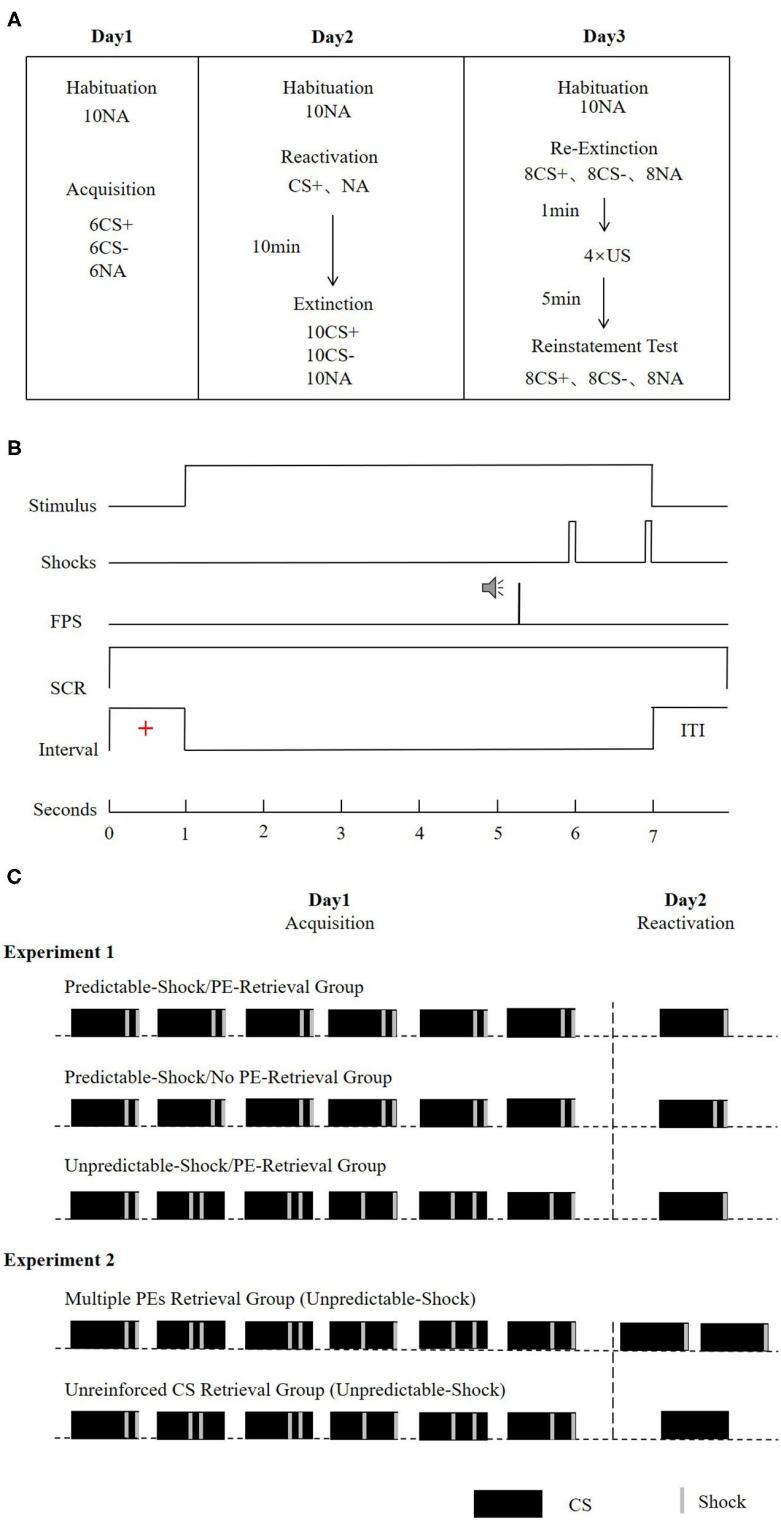
**(A)** Schematic representation of the experimental design. During extinction, re-extinction, and re-instatement test, all CSs were presented without reinforcement. **(B)** Conditioning trials of a reinforced CS+ presentation. In the CS- and the unreinforced CS+ trials, no US was delivered. **(C)** Reinforcement schedule of the CS+ during the acquisition and reactivation phase for the experimental groups. For the predictable-shock acquisition conditions, each CS+ was paired with two shocks that occurred in fixed time. For the unpredictable-shock acquisition conditions, each CS+ was paired with two shocks that occurred pseudorandomly during the stimulus. The prediction error is defined by the interaction between the information in the reactivation session and the learning history. In the acquisition, participants learned that every CS+ was followed by two shocks (100% reinforcement schedule). In the reactivation, the no PE retrieval condition was manipulated as a single reactivation trial paired with two shocks (consistent with expectations). The PE retrieval condition involved a single reactivation trial paired with only one shock (violation of expectation). Furthermore, the increase of PE was manipulated as two reactivation trials (each paired with a shock, as in the multiple PEs retrieval group) or a single non-reinforced reactivation trial (the unreinforced CS retrieval group).

The entire experiment was carried out over the course of three successive days, with the modules separated by about 24 h. All the subjects arrived at the lab between 9:00 and 18:00 h and were instructed not to nap after the experimental sessions. Regular wake and sleep patterns were maintained on the experimental days. During each experimental module, the subjects sat 50 cm facing a computer screen on a table in a sound-attenuated room. We started each of the session with a 1-min acclimatization window comprising 70-dB broadband noise, which was continued to the end of the module information of background noise. This was succeeded by a habituation stage, which was composed of 10 startle probes to decrease the initial startle reactivity. The attributes of the CSs, ITIs, trial order, as well as the startle probes in the reactivation-extinction (day 2), and the re-extinction (day 3) tests were comparable with those in the acquisition (day 1). A schematic illustration of the experimental design is described in Figure 1A.

#### Day 1: Acquisition

The subjects sat facing a personal computer with EMG, SCR, as well as US-electrodes linked. Before conditioning learning, the uncomfortable but not painful shock strength was determined for each subject. The protocol was started with 10 habituation trials for stabilizing the general startle responding. In the conditioning training, CS+ was paired with two shocks, whereas CS– was never paired with shocks. The subjects were notified that a shock in the trials will follow one of the stimuli, while others would not be followed by the shock. Acquisition constituted six presentations for each CS, as well as six noise-alone presentations. We presented both CSs for 6 s, and the startle probe was conveyed 4,300 ms following the onset of CS. The order of different types of trials (i.e., CS+, CS–, and NA) was pseudorandomized, with the condition that the 1^st^ trial not be followed by shocks and that no more than two stimuli of the same kind be presented consecutively. Intertrial intervals (ITI) fluctuated between 15, 16, and 17 s with a mean of 16 s (Figure 1B). The trial procedure is largely based on van Dis et al. (2019) and Yang et al. (2019). FPS has a short onset latency (21–120 ms for acoustically elicited blinks), while the latency of SCR is at least 1 s (more extended responses could be theoretically possible) (Blumenthal et al., 2005; Christopoulos et al., 2016). Assuming that the startle probe would elicit SCR, it also occurred 5 s after CS onset (beyond our SCR analysis window). Hence, we believe that SCR and FPS do not affect each other.

In the standard acquisition condition (predictable-shock/PE-retrieval group and predictable-shock/no PE-retrieval group in experiment 1), the US occurrence time of each CS+ was the same and was administered at 4,800 and 5,800 ms after CS+ onset. In the enhancement acquisition condition (unpredictable-shock/PE-retrieval group in experiment 1 and two groups in experiment 2), shocks were presented at 4,800 and 5,800 ms after the first CS+ onset, at 2,800 and 3,800 ms after the second CS+ onset, at 3,800 and 4,800 ms after the third CS+ onset, at 2,800 and 5,800 ms after the fourth CS+ onset, at 2,800 and 4,800 ms after the fifth CS+ onset, and at 3,800 and 5,800 ms after the sixth CS+ onset (Amadi et al., 2017) (Figure 1C). To prevent the influence of electric shocks on SCR, the peak within the 1–3-s window after the onset of the stimulus was used in the SCR analyses. Furthermore, subjects were pointedly informed to recall what they had acquired in the acquisition. These directions were set to promote retention of the CS-US contingency on the successive days (Norrholm et al., 2006) and to impede the subjects from incorrectly expecting a dissimilar contingency scheme during ensuing testing.

#### Day 2: Reactivation Sessions and Extinction

Reactivation of memory occurred 24 h following fear learning. The subjects were granted a 10-min to watch an excerpt called Planet Earth from a documentary by the British Broadcasting Corporation (Li et al., 2017). Extinction learning followed immediately and consisted of 10 NA presentations and 10 CS presentations, which were not followed by electric shocks.

Experiment 1: Reactivation included 1 CS+ and 1 NA. In the PE retrieval condition (predictable-shock/PE-retrieval group and unpredictable-shock/PE-retrieval group), an electric shock was presented at 5,800 ms after CS+ onset, lasted for 200 ms, and disappeared together with CS+. In the No-PE retrieval condition (predictable-shock/no PE-retrieval group), two electric shocks were administered at 4,800 and 5,800 ms after CS+ onset, and the procedure is the same as that in acquisition (Figure 1C).

Experiment 2: In the reactivation sessions, subjects in the multiple PE retrieval group underwent two CS+ for retrieval, with an electric shock presented at 5,800 ms after each CS+ onset. In addition, subjects in the unreinforced CS retrieval group underwent just one unreinforced CS+ for retrieval (Figure 1C).

#### Day 3: Re-extinction and Reinstatement Test

Subjects underwent tests of instinctive recovery, as well as reinstatement of fear 24 h following postretrieval extinction. During the re-extinction stage, we exposed the subjects to the CS+, as well as CS– for eight times without the US, and eight startle probes were delivered alone (NA). Postre-extinction, the subjects were presented with four unsignaled USs. The time between the last re-extinction trial and the reinstating USs was 1 min. After the unsignaled US, subjects took a 5-min break and were then presented with eight unreinforced CS+, CS–, and NA trials (reinstatement test).

### Statistical Analyses

For both FPS and SCR data, the primary dependent variable was the mean differential (md), which was the response to the CS+ in each trial less the responses to the CS–, and then averaged across the subjects. The md FPS and md SCR underwent a mixed analysis of variance (ANOVA) for repeated measures between factors of groups and within-subject factors of time (early and late phase).

Paired samples *t*-tests of the differential responses in the last half of the acquisition were employed to estimate the fear acquisition, while comparisons of the differential responses of the last trial of extinction were utilized to explore extinction. The spontaneous recovery was examined *via* the fear recovery index: difference in the differential responses from last trial of extinction to the first trial of re-extinction. The reinstatement index was determined by evaluating the differential responses from the last trial of re-extinction to the first trial of the reinstatement test stage. Thereafter, one-way ANOVA between groups of differential responses of the first trial of spontaneous recovery and the first trial of reinstatement test were conducted respectively, to estimate the comparable superiority of each to impede the return of fear.

We performed the *post-hoc* tests *via* the Fisher's LSD between groups. *P* < 0.05 signified statistical significance and reported partial η^2^ indicated the estimate of effect size. We employed the Greenhouse-Geisser adjustments of degrees of freedom when appropriate.

## Results

### Experiment 1

#### Fear-Potentiated Startle

**Figure 2:**
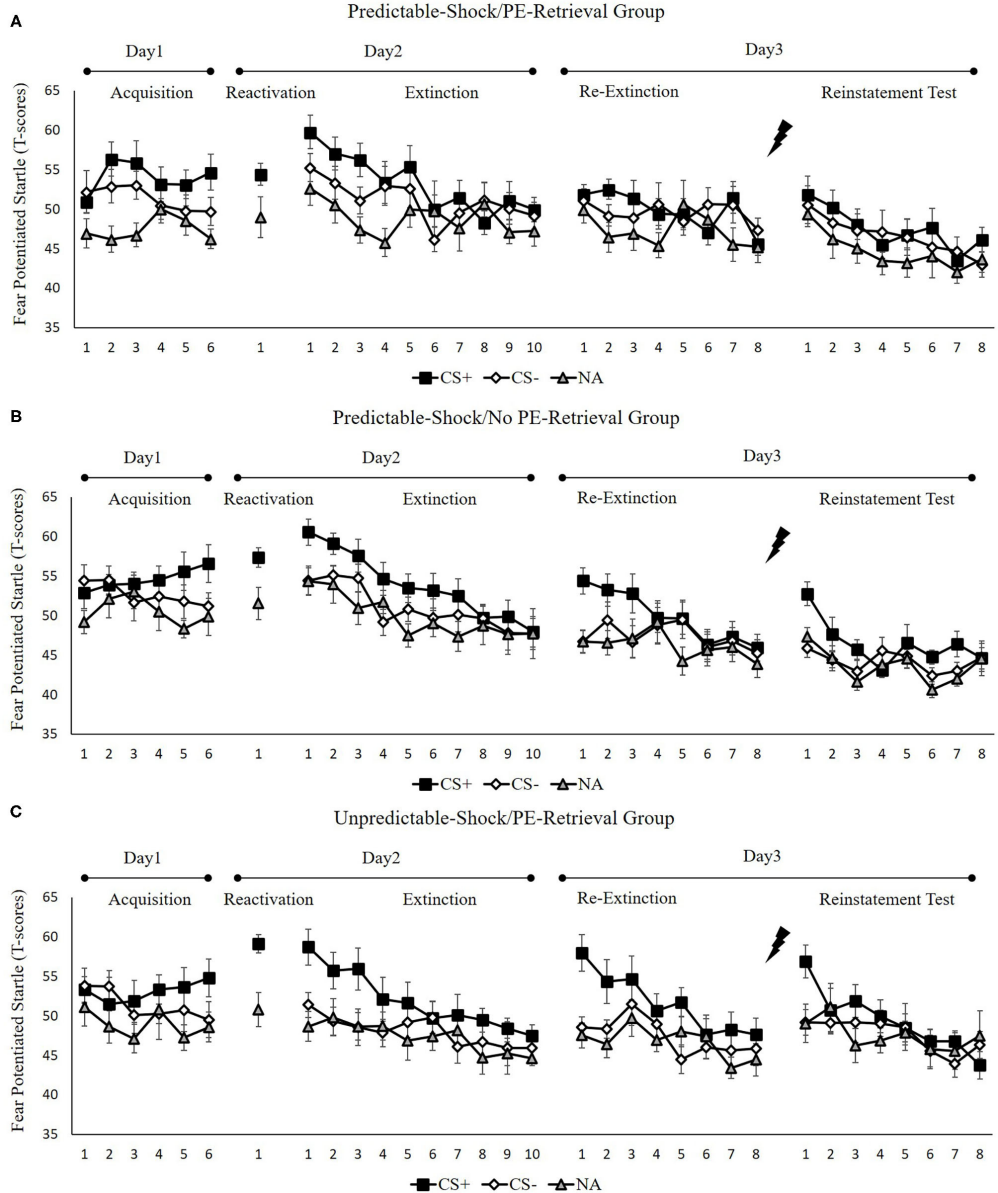
Mean startle potentiation to the fear-conditioned stimulus (CS+), the control stimulus (CS–), and the noise-alone (NA) trials during acquisition, memory reactivation, extinction, re-extinction, and reinstatement test for **(A)** the predictable-shock/PE-retrieval group (*n* = 18), **(B)** the predictable-shock/no PE-retrieval group (*n* = 19), and **(C)** the unpredictable-shock/PE-retrieval group (*n* = 16). Error bars represent SEM.

Figure 2 indicates the mean fear-potentiated startle responses per trial for each group, across all experimental days and phase.

##### Fear Acquisition and Post-retrieval Extinction

We conducted mixed ANOVAs with a between-subject factor of groups and within-factor of the time (early and late phase) and stimulus type to examine the conditioned fear acquisition, as well as extinction. In acquisition, a considerable stimulus × time interplay [*F*(1, 50) = 7.77, *p* < 0.01, $\eta_{p}^{2}$ = 0.14] was reported but no marked interplay of the group × stimulus [*F*(2, 50) = 0.18, *p* = 0.84] or the group× time [*F*(2, 50) = 0.73, *p* = 0.49]. Besides, there was no remarkable interplay of the three factors [*F*(2,50) = 0.32, *p* = 0.72]. The correlated pairs *t*-test of the last three trials of acquisition exhibited a remarkably elevated FPS to CS+ in contrast with CS– [*t*(52) = 3.99, *p* < 0.001, *d* = 0.55]. These data demonstrated that subjects developed a conditioned fear to the CS+ and not to the CS–. In extinction, there was a distinct interplay between the stimulus × time [*F*(1, 50) = 18.51, *p* < 0.001, $\eta_{p}^{2}$ = 0.27] but no remarkable interplay of the group × stimulus [*F*(2, 50) = 1.54, *p* = 0.23] or the group × time [*F*(2, 50) = 0.22, *p* = 0.80]. Moreover, there were no remarkable interplay of the three factors [*F*(2, 50) = 0.25, *p* = 0.78]. A correlated pairs *t*-test of the last trial of extinction exhibited no marked difference in FPS to CS+ relative to the CS– [*t*(52) = 1.40, *p* = 0.17]. These data show that extinction alleviated discrimination between the CS+ and CS–.

To measure the difference of fear memory strength caused by predictable-shock and unpredictable-shock acquisition, we examined the FPS of CS+ from the last trial of the acquisition to the trial of reactivation. There was no marked primary outcome of trials [*F*(2, 50) = 0.82, *p* = 0.45]. However, we observed a remarkable primary effect of group during reactivation [*F*(2, 50) = 3.41, *p* < 0.05, $\eta_{p}^{2}$ = 0.12]. Moreover, one-way ANOVA between three groups of the early phase of extinction of the differential FPS revealed a distinct primary effect of the group [*F*(2, 50) = 3.26, *p* < 0.05, $\eta_{p}^{2}$ = 0.12]. *Post-hoc* assessment exhibited remarkable difference between the predictable-shock/PE-retrieval group and the unpredictable-shock/PE-retrieval group (*p* < 0.05); the predictable-shock/no PE-retrieval group and the unpredictable-shock/PE-retrieval group (*p* < 0.05), whereas no difference was reported between the predictable-shock/PE-retrieval group and the predictable-shock/no PE-retrieval group.

##### Test Performance

**Figure 4A** indicates the mean differential FPS of the key trials of spontaneous recovery, as well as reinstatement test in three groups.

*Spontaneous recovery*. A mixed ANOVA with groups, as well as trials (last trial of extinction, first trial of re-extinction) exhibited a remarkable primary outcome of trials [*F*(1, 50) = 20.79, *p* < 0.001, $\eta_{p}^{2}$ = 0.29] and groups [*F*(2, 50) = 5.76, *p* < 0.01, $\eta_{p}^{2}$ = 0.19] and an interplay of both factors [*F*(2, 50) = 5.27, *p* < 0.01, $\eta_{p}^{2}$ = 0.17].

To examine the spontaneous fear recovery, the md FPS between the last trial of extinction and the first trial of re-extinction in each group were compared. A paired samples *t*-test exhibited no marked difference only in the predictable-shock/PE-retrieval group [*t*(17) = 0.01, *p* = 0.99], but remarkable difference were revealed in the predictable-shock/no PE-retrieval group [*t*(18) = −3.79, *p* < 0.01, *d* = −0.87] and the unpredictable-shock/PE-retrieval group [*t*(15) = −3.17, *p* < 0.01, *d* = −0.79].

One-way ANOVA of the first trial of re-extinction of the differential FPS between CS+ and CS– indicated a remarkable primary effect of the groups [*F*(2, 50) = 7.64, *p* < 0.01, $\eta_{p}^{2}$ = 0.23]. The *post-hoc* evaluation exhibited a distinct difference between the predictable-shock/PE-retrieval group and the predictable-shock/no PE-retrieval group (*p* < 0.05) and the predictable-shock/PE-retrieval group and the unpredictable-shock/PE-retrieval group (*p* < 0.01). No differences between the predictable-shock/no PE-retrieval group and the unpredictable-shock/PE-retrieval group were detected.

These results suggest that the three groups show different levels of FPS in spontaneous recovery. Specifically, the predictable-shock/PE-retrieval group exhibited the lowest levels of FPS response, whereas the predictable-shock/no PE-retrieval group, as well as the unpredictable-shock/PE-retrieval group exhibited remarkably higher levels of FPS response.

*Reinstatement*. A mixed ANOVA with groups, as well as trials (last trial of re-extinction, first trial of reinstatement) exhibited a marked primary outcome of trials [*F*(1, 50) = 15.93, *p* < 0.001, $\eta_{p}^{2}$ = 0.24] and groups [*F*(2, 50) = 7.01, *p* < 0.01, $\eta_{p}^{2}$ = 0.22] but no distinct interaction effect of both factors [*F*(2,50) = 0.61, *p* = 0.55].

To examine the fear reinstatement, the md FPS between the last trial of re-extinction and the first trial of reinstatement were compared in each group. Consequently, the paired samples *t*-test exhibited no remarkable difference in the predictable-shock/PE-retrieval group [*t*(17) = −1.70, *p* = 0.11], but there was marked difference in the predictable-shock/no PE-retrieval group [*t*(18) = −3.22, *p* < 0.01, *d* = −0.74] and the unpredictable-shock/PE-retrieval group [*t*(15) = −2.07, *p* < 0.05, *d* = −0.52].

One-way ANOVA between three groups of the first trial of reinstatement of the differential FPS revealed a remarkable primary effect of the groups [*F*(2, 50) = 4.67, *p* < 0.05, $\eta_{p}^{2}$ = 0.16]. *Post-hoc* assessment exhibited a marked difference between the predictable-shock/PE-retrieval group and the predictable-shock/no PE-retrieval group (*p* < 0.05) and the predictable-shock/PE-retrieval group and the unpredictable-shock/PE-retrieval group (*p* < 0.01). No difference between the predictable-shock/no PE-retrieval group and the unpredictable-shock/PE-retrieval group was detected.

These results suggest that three groups show difference levels of FPS in reinstatement. Similar to spontaneous recovery, the predictable-shock/PE-retrieval group exhibited the lowest levels of FPS response, whereas the predictable-shock/no PE-retrieval group, as well as the unpredictable-shock/PE-retrieval group exhibited remarkably higher levels of FPS response.

#### Skin Conductance Response

**Figure 3:**
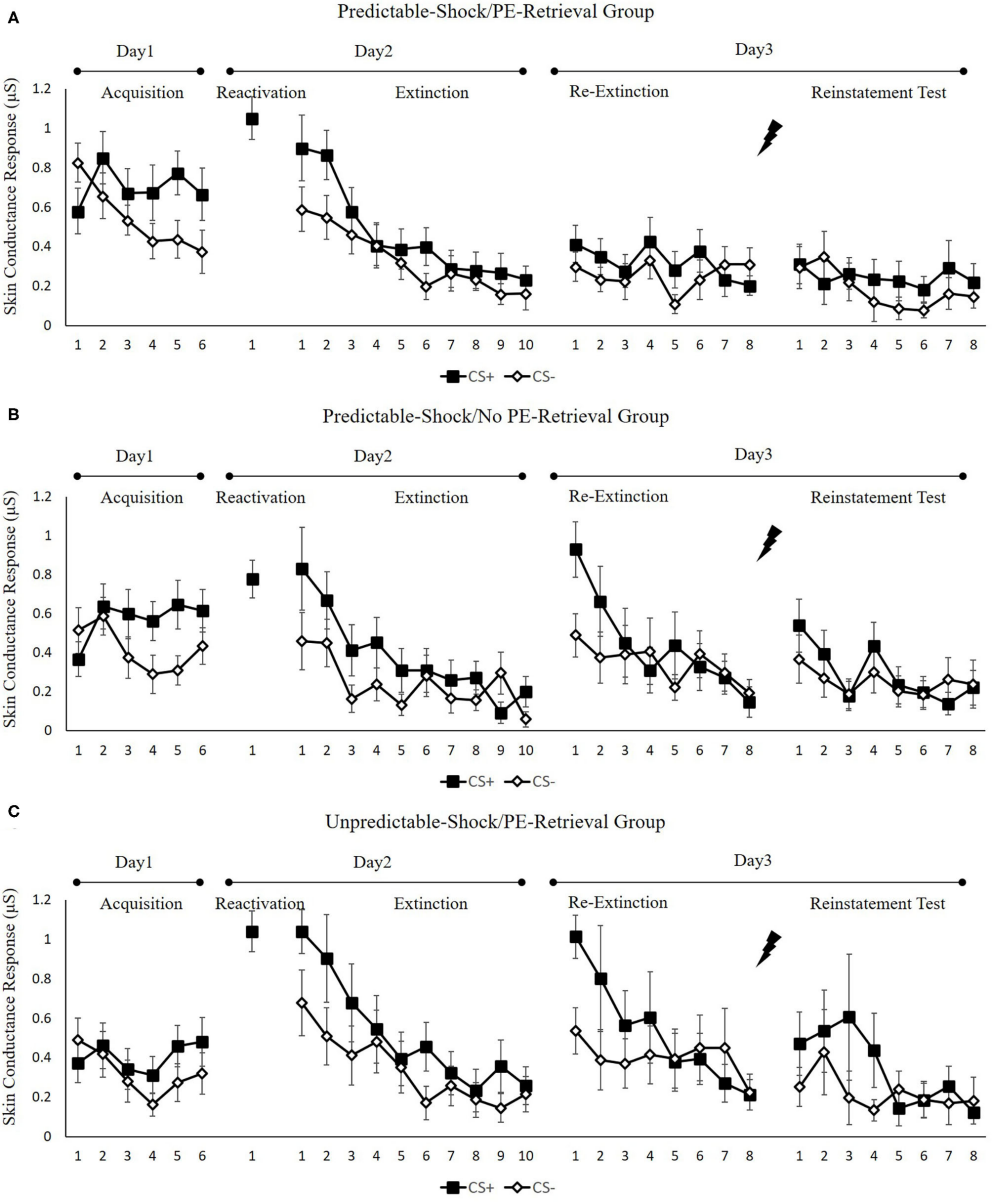
Mean skin conductance responses to the CS+ and CS– trials during acquisition, memory reactivation, extinction, re-extinction, and reinstatement test for **(A)** the predictable-shock/PE-retrieval group (*n* = 18), **(B)** the predictable-shock/no PE-retrieval group (*n* = 19), and **(C)** the unpredictable-shock/PE-retrieval group (*n* = 16). Error bars represent SEM.

Figure 3 indicates the mean skin conductance responses per trial for each group, across all experimental days, as well as phases.

##### Fear Acquisition and Post-retrieval Extinction

We conducted mixed ANOVAs with a between-subject factor of groups and within-factor of the time (early and late phase) and stimulus type to assess conditioned fear acquisition, as well as extinction. In acquisition, there was a remarkable stimulus × time interaction [*F*(1, 50) = 27.44, *p* < 0.01, $\eta_{p}^{2}$ = 0.35] but no remarkable interplay of the group × stimulus [*F*(2, 50) = 0.52, *p* = 0.60] or the group× time [*F*(2, 50) = 0.87, *p* = 0.43]. Additionally, there was no marked interplay of the three factors [*F*(2, 50) = 0.42, *p* = 0.66]. The paired samples *t*-test of the last three trials of acquisition exhibited a remarkable elevated SCR to CS+ relative to CS– [*t*(52) = 5.59, *p* < 0.001, *d* = 0.77]. These data exhibited that subjects developed a conditioned fear to the CS+ and not to the CS–. In extinction, there was a remarkable interplay between the stimulus × time [*F*(1, 49) = 9.48, *p* < 0.01, $\eta_{p}^{2}$ = 0.16] but not marked interplay of the group × stimulus [*F*(2, 49) = 0.36, *p* = 0.70] or the group × time [*F*(2, 49) = 1.05, *p* = 0.36]. There was also no remarkable interplay of the three factors [*F*(2, 49) = 1.26, *p* = 0.29]. A correlated samples *t*-test of the last trial of extinction show no remarkable difference in SCR to CS+ in contrast with CS– [*t*(50) = 1.92, *p* = 0.06]. These data demonstrated that extinction abrogated discrimination between the CS+ and CS–.

To measure the difference of fear memory strength caused by predictable-shock and unpredictable-shock acquisition, we examined the SCR of CS+ from the last trial of the acquisition to the trial of reactivation. A remarkable primary outcome of trials [*F*(1, 44) = 14.85, *p* < 0.01, $\eta_{p}^{2}$ = 0.25] was reported. A correlated samples *t*-test of different SCR between the last trial of acquisition and the retrieval trial in each group exhibited a remarkable increase in the unpredictable-shock/PE-retrieval group [*t*(15) = −4.19, *p* < 0.001, *d* = −1.05] but not in the predictable-shock/PE-retrieval group [*t*(14) = −1.87, *p* = 0.08] and the predictable-shock/no PE-retrieval group [*t*(15) = −0.823, *p* = 0.42].

##### Test Performance

**Figure 4:**
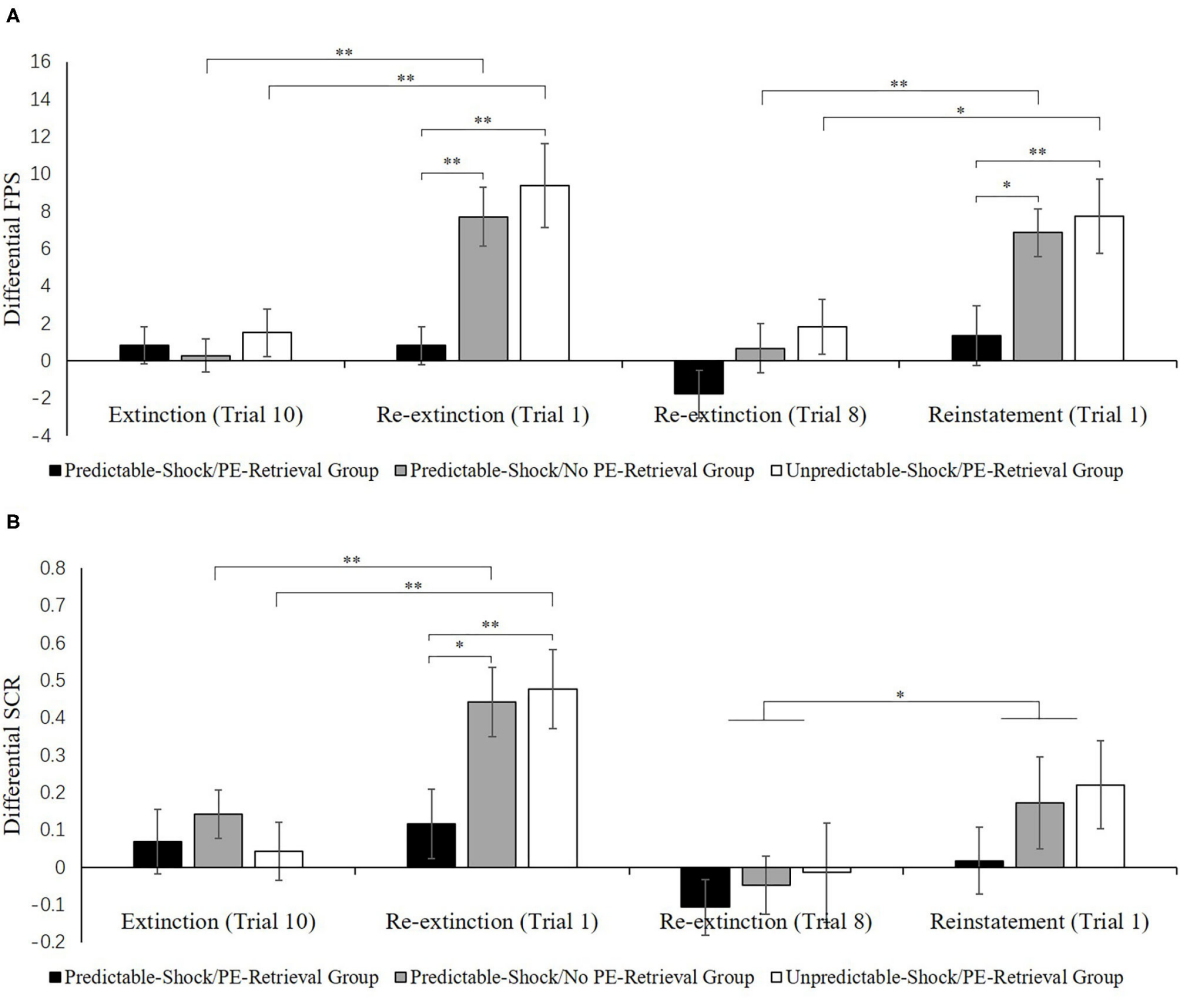
Mean differential FPS **(A)** and SCR **(B)** (CS+ minus CS–) during extinction (last trial), re-extinction (first trial; last trial), and reinstatement (first trial) for the experiment 1. Error bars represent SEM. *p < 0.05; **p < 0.01.

Figure 4B indicates the mean differential SCR of the key trials of spontaneous recovery, as well as reconsolidation test in three groups.

*Spontaneous recovery*. A mixed ANOVA with groups and trials (last trial of extinction, first trial of re-extinction) revealed a remarkable primary outcome of trials [*F*(1, 50) = 12.72, *p* < 0.001, $\eta_{p}^{2}$ = 0.20] and groups [*F*(2, 50) = 3.32, *p* < 0.05, $\eta_{p}^{2}$ = 0.12] but no remarkable interaction effect of both factors[*F*(2, 50) = 2.27, *p* = 0.11].

To examine the spontaneous fear recovery, the md SCR between the last trial of extinction and the first trial of re-extinction in each group was compared. The correlated samples *t*-test exhibited no remarkable difference only in the predictable-shock/PE-retrieval group [*t*(17) = −0.34, *p* = 0.74], but a remarkable difference was revealed in the predictable-shock/no PE-retrieval group [*t*(18) = −3.14, *p* < 0.01, *d* = −0.72] and the unpredictable-shock/PE-retrieval group [*t*(15) = −3.25, *p* < 0.01, *d* = −0.81].

One-way ANOVA of the first trial of re-extinction of the differential SCR exhibited a remarkable primary outcome of the groups [*F*(2, 50) = 4.22, *p* < 0.05, $\eta_{p}^{2}$ = 0.14]. *Post-hoc* analysis exhibited a remarkable difference between the predictable-shock/PE-retrieval group and the predictable-shock/no PE-retrieval group (*p* < 0.05) and the predictable-shock/PE-retrieval group and the unpredictable-shock/PE-retrieval group (*p* < 0.01). No differences between the predictable-shock/no PE-retrieval group and the unpredictable-shock/PE-retrieval group were detected.

In line with the FPS, these results suggest that three groups show difference levels of SCR in spontaneous recovery. Specifically, the predictable-shock/PE-retrieval group had the lowest levels of SCR, whereas the predictable-shock/no PE-retrieval group and the unpredictable-shock/PE-retrieval group had remarkably higher levels of SCR.

*Reinstatement*. A mixed ANOVA with groups, as well as trials (last trial of re-extinction, first trial of reinstatement) only exhibited a remarkable primary outcome of trials [*F*(1, 50) = 4.46, *p* < 0.05, $\eta_{p}^{2}$ = 0.08] but no remarkable primary outcome of groups [*F*(2, 50) = 1.27, *p* = 0.29] and interaction outcome of both factors [*F*(2, 50) = 0.14, *p* = 0.87]. A correlated samples *t*-test of the md SCR from the last trial of re-extinction to the first trial of reinstatement exhibited no remarkable increase in each group [*t*s < −0.97, *p*s > 0.13, *d*s < −0.23]. As expected, one-way ANOVA of the first trial of reinstatement of the differential SCR revealed no remarkable primary outcome of the groups [*F*(2, 50) = 0.89, *p* = 0.42]. These results suggest that the three groups do not show any difference levels of SCR in reinstatement, which were inconsistent with FPS.

### Experiment 2

#### Fear-Potentiated Startle

**Figure 5:**
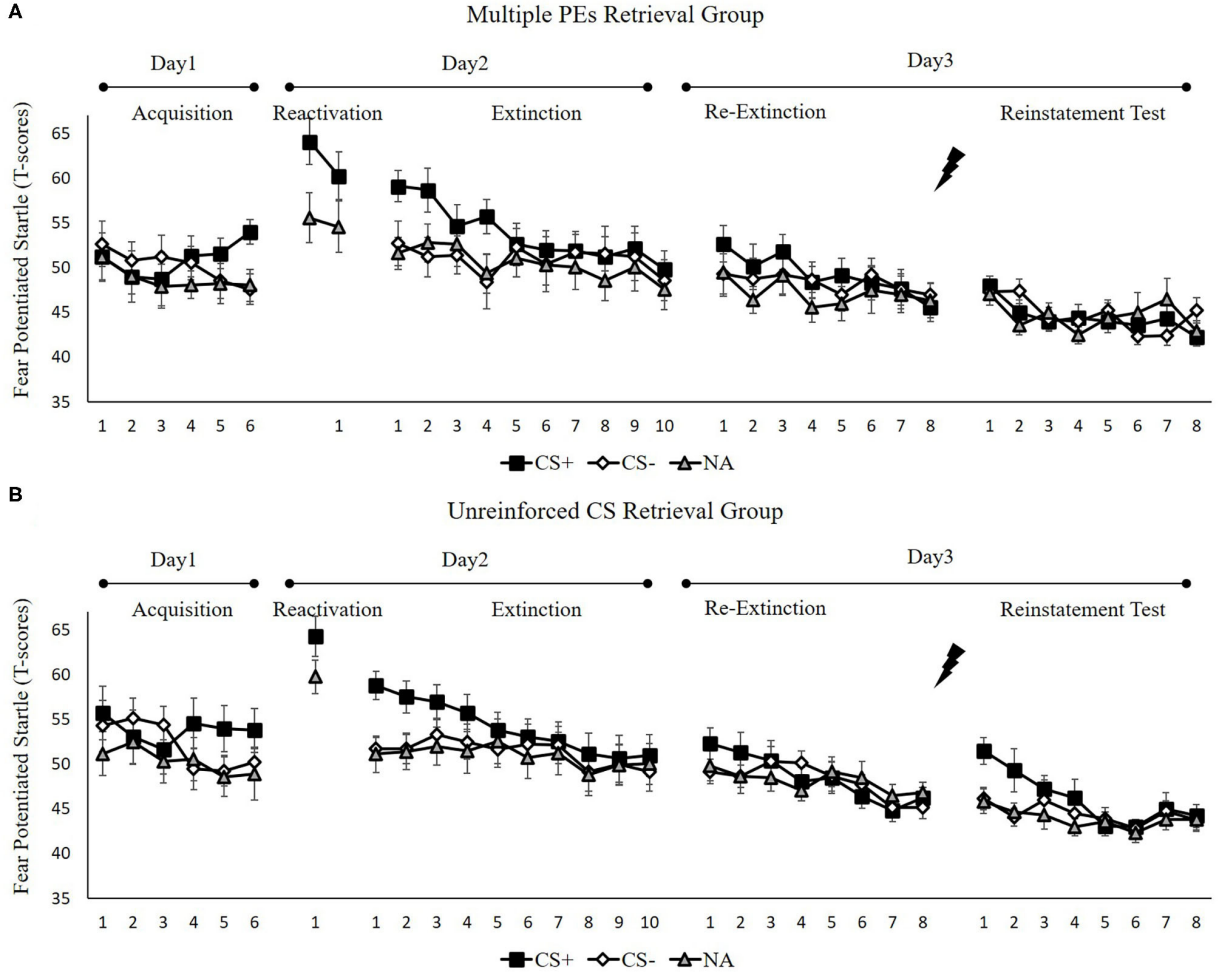
Mean startle potentiation to the fear conditioned stimulus (CS+), the control stimulus (CS–), and the noise-alone (NA) trials during acquisition, memory reactivation, extinction, re-extinction, and reinstatement test for **(A)** the multiple PE retrieval group (*n* = 17) and **(B)** the unreinforced CS retrieval group (*n* = 17). Error bars represent SEM.

Figure 5 shows the mean fear-potentiated startle responses per trial for each group, across all experimental days and phases.

##### Fear Acquisition and Post-retrieval Extinction

We conducted mixed ANOVAs with a between-subject factor of groups and within-factor of the time (early and late phase) and stimulus type to assess conditioned fear acquisition and extinction. In acquisition, a remarkable stimulus × time interaction [*F*(1, 32) = 14.08, *p* < 0.001, $\eta_{p}^{2}$ = 0.31] but not remarkable interplay of the group × stimulus [*F*(1, 32) = 0.21, *p* = 0.65] or the group × time [*F*(1, 32) = 2.08, *p* = 0.16] were reported. Moreover, no remarkable interplay of the three factors [*F*(1, 32) = 0.01, *p* = 0.92] was reported. A correlated samples *t*-test of the last three trials of acquisition exhibited a remarkable higher FPS to CS+ relative to the CS– [*t*(33) = 4.14, *p* < 0.001, *d* = 0.71]. These data demonstrated that subjects developed a conditioned fear to the CS+ and not to the CS–. In extinction, there was a remarkable interplay between the stimulus × time [*F*(1, 32) = 17.57, *p* < 0.001, $\eta_{p}^{2}$ = 0.35] but not remarkable interplay of the group × stimulus [*F*(1, 32) = 0.09, *p* = 0.97] or the group × time [*F*(1, 32) = 0.18, *p* = 0.68]. Additionally, no remarkable interplay of the three factors [*F*(1, 32) = 0.31, *p* = z0.58] was reported. A correlated samples *t*-test of the last trial of extinction show no remarkable difference in FPS to CS+ than to CS– [*t*(33) = 1.80, *p* = 0.08]. These data demonstrated that extinction abrogated discrimination between the CS+ and CS–.

To ensure that the two groups of subjects produce enhance fear by unpredictable-shock acquisition, we examined the FPS of CS+ from the last trial of the acquisition to the first trial of reactivation. A remarkable primary effect of trials [*F*(1, 32) = 20.57, *p* < 0.001, $\eta_{p}^{2}$ = 0.39] was observed. A paired samples *t*-test exhibited a remarkable increase FPS to CS+ from the last trial of acquisition to the first retrieval trial [*t*(33) = −4.61, *p* < 0.001, *d* = −0.79], similar to unpredictable-shock/PE-retrieval group in experiment 1. Moreover, one-way ANOVA of the early phase of extinction of the differential FPS revealed no remarkable primary outcome of the group [*F*(1, 32) = 0.01, *p* = 0.93].

##### Test Performance

**Figure 7A** shows the mean differential FPS of the key trials of spontaneous recovery, as well as the reconsolidation test in two groups.

*Spontaneous recovery*. A mixed ANOVA with groups, as well as the trials (last trial of extinction, first trial of re-extinction) revealed no significance in all primary outcomes and interactions [*F* < 1.98, *p* > 0.17, $\eta_{p}^{2}$ < 0.05]. A dependent samples *t*-test of the md FPS from the last trial of extinction to the first trial of re-extinction exhibited no remarkable increase in each group [*t* < −0.74, *p* > 0.23, *d* < −0.18]. As expected, one-way ANOVA of the first trial of re-extinction of the differential FPS revealed no remarkable primary outcome of the groups [*F*(1, 32) = 0.01, *p* = 0.93]. These data demonstrate that both groups have prevented the increase levels of SCR in spontaneous recovery.

*Reinstatement*. A mixed ANOVA with groups, as well as trials (last trial of re-extinction, first trial of reinstatement) exhibited a remarkable primary outcome of trials [*F*(1, 32) = 7.82, *p* < 0.01, $\eta_{p}^{2}$ = 0.20] and groups [*F*(1, 32) = 6.08, *p* < 0.02, $\eta_{p}^{2}$ = 0.16] but no remarkable interaction outcome of both factors[*F*(1, 32) = 1.00, *p* = 0.32].

To explore the reinstatement of fear, the md FPS between the last trial of re-extinction and the first trial of reinstatement was compared in each group. A correlated pairs *t*-test exhibited no remarkable difference in the multiple PE retrieval group [*t*(16) = −1.28, *p* = 0.22], but remarkable difference existed in the unreinforced CS retrieval group [*t*(16) = −2.67, *p* < 0.05, *d* = −0.65]. One-way ANOVA between groups of the first trial of reinstatement of the differential FPS revealed a remarkable primary outcome of the groups [*F*(1, 32) = 7.41, *p* < 0.05, $\eta_{p}^{2}$ = 0.19].

These results suggest that two groups show difference levels of FPS in reinstatement. The unreinforced CS retrieval group had remarkably higher levels of FPS response than the multiple PE retrieval group.

#### Skin Conductance Response

**Figure 6:**
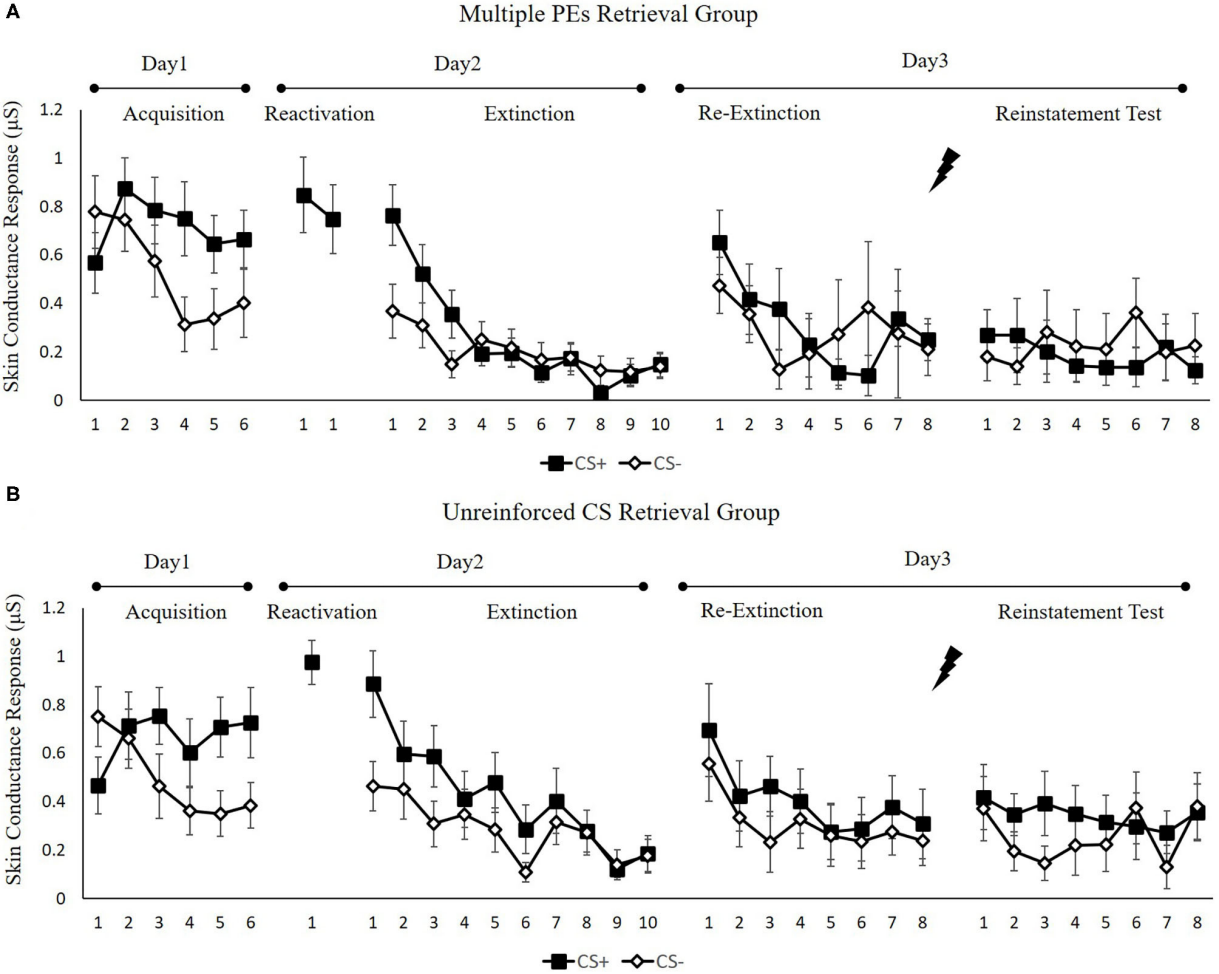
Mean skin conductance responses to the CS+ and CS– trials during acquisition, memory reactivation, extinction, re-extinction, and reinstatement test for **(A)** the multiple PE retrieval group (*n* = 17) and **(B)** the unreinforced CS retrieval group (*n* = 17). Error bars represent SEM.

Figure 6 shows the mean skin conductance responses per trial for each group, across all experimental days and phases.

##### Fear Acquisition and Post-retrieval Extinction

We conducted mixed ANOVAs with a between-subject factor of groups and within-factor of the time (early and late phase) and stimulus type to assess conditioned fear acquisition and extinction. In acquisition, a remarkable stimulus × time interaction [*F*(1, 32) = 20.89, *p* < 0.001, $\eta_{p}^{2}$ = 0.40] but no remarkable interplay of the group × stimulus [*F*(1, 32) = 0.09, *p* = 0.76] or the group × time [*F*(1, 32) = 1.25, *p* = 0.27] were reported. Moreover, no remarkable interplay of the 3 factors [*F*(1,32) = 0.08, *p* = 0.97] was observed. A dependent samples *t*-test of the last three trials of acquisition exhibited a remarkable elevated SCR to CS+ relative to CS– [*t*(33) = 4.99, *p* < 0.001, *d* = 0.86]. These data demonstrated that subjects developed a conditioned fear to the CS+ and not the CS–. In extinction, there was a remarkable interplay between the stimulus × time [*F*(1, 32) = 14.73, *p* < 0.001, $\eta_{p}^{2}$ = 0.32] but not remarkable interplay of the group × stimulus [*F*(1, 32) = 4.54, *p* = 0.70] or the group × time [*F*(1, 32) = 0.49, *p* = 0.48]. Besides, no remarkable interplay of the three factors [*F*(1, 32) = 0.02, *p* = 0.90] was reported. A repeated measures *t*-test of the last trial of extinction exhibited no remarkable difference in SCR to CS+ relative to the CS– [*t*(33) = 0.32, *p* = 0.83]. These data demonstrated that extinction abrogated discrimination between the CS+ and CS–.

To ensure that the two groups of subjects fear enhanced by unpredictable-shock acquisition, we examined the SCR of CS+ from the last trial of the acquisition to the first trial of reactivation. A remarkable primary outcome of trials [*F*(1, 32) = 4.36, *p* < 0.05, $\eta_{p}^{2}$ = 0.12] was observed. A repeated measures t-test exhibited a remarkable increase SCR to CS+ from the last trial of acquisition to the first retrieval trial [*t*(33) = −2.12, *p* < 0.05, *d* = −0.36]. Moreover, one-way ANOVA of the early phase of extinction of the differential SCR revealed no remarkable primary outcome of the group [*F*(1, 32) = 0.01, *p* = 0.92].

##### Test Performance

**Figure 7:**
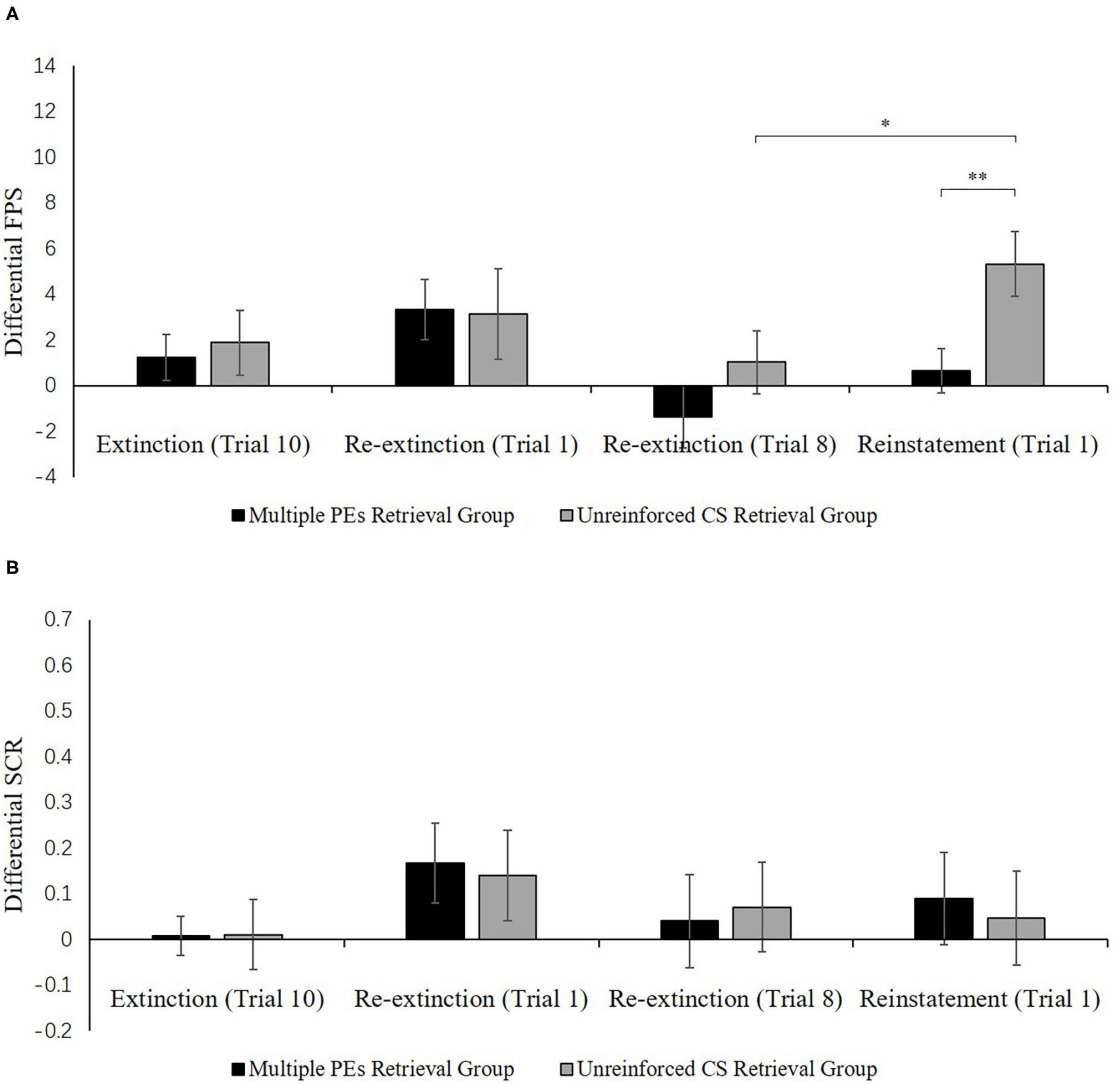
Mean differential FPS **(A)** and SCR **(B)** (CS+ minus CS–) during extinction (last trial), re-extinction (first trial; last trial), and reinstatement (first trial) for the experiment 2. Error bars represent SEM. *p < 0.05; **p < 0.01.

Figure 7B shows the mean differential SCR of the key trials of spontaneous recovery, as well as reconsolidation test in two groups.

*Spontaneous recovery*. A mixed ANOVA with groups, as well as trials (last trial of extinction, first trial of re-extinction) exhibited no significance in all primary outcomes and interactions [*F* < 2.61, *p* > 0.11, $\eta_{p}^{2}$ < 0.07]. A paired samples *t*-test of the md SCR from the last trial of extinction to the first trial of re-extinction exhibited no remarkable increase in each group [*t* < −0.86, *p* > 0.11, *d* < −0.21]. As expected, one-way ANOVA of the first trial of re-extinction of the differential SCR revealed no remarkable primary outcome of the groups [*F*(1, 32) = 0.04, *p* = 0.84]. These results suggest that both two groups have prevented the increase levels of SCR in spontaneous recovery.

*Reinstatement*. A mixed ANOVA with groups, as well as trials (last trial of re-extinction, first trial of reinstatement) exhibited no significance in all primary outcomes and interactions [*F* < 0.22, *p* > 0.64, $\eta_{p}^{2}$ < 0.01]. A dependent samples t-test of the md SCR from the last trial of re-extinction to the first trial of reinstatement exhibited no remarkable increase in each group [*t* < 0.22, *p* > 0.66, *d* < 0.05]. As expected, one-way ANOVA of the first trial of reinstatement of the differential SCR revealed no remarkable primary outcome of the groups [*F*(1, 32) = 0.09, *p* = 0.77]. These results suggest that in both groups, the increase in the levels of SCR in the reinstatement test was prevented, which were inconsistent with FPS.

## Discussion

In this study, we found that applied extinction training 10 min postretrieval did not prevent fear-potentiated startle, as well as skin conductance responses in spontaneous recovery, and reinstatement tests, when the parameters of acquisition and retrieval were identical (no PE). On the other hand, retrieval-extinction decreased spontaneous recovery (FPS and SCR) and reinstatement of fear (FPS) when a mismatch occurred between initial training and memory retrieval (PE). Our findings indicate that PE is necessary to destabilize consolidated memory in the behavioral reconsolidation inference procedure. The most important finding in this study is that the degree of PE needed to induce memory destabilization during memory retrieval may depend on the strength of fear memory. In other words, the sufficient degrees of PE needed to destabilize ordinary fear memory are insufficient to destabilize enhanced fear memory. In our study, the sufficient degree of PE for ordinary fear memory destabilization was single PE (one CS was paired with one shock), while that for destabilization of enhanced fear memory was multiple PE (two consecutive CSs in which each paired with one shock).

In human fear conditioning research, many methods have been used to measure conditioned responses, including skin conductance, startle electromyography, pupillometry, US expectancy, and valence ratings (Lonsdorf et al., 2017; Zuccolo and Hunziker, 2019). These measures could be sensitive to different aspects of learning and memory. For example, FPS is an indirect measure of fear modulated by emotional valence (it is enhanced during negative and reduced during positive). In contrast, SCR reflects peripheral autonomic responses, which is a valence-unspecific measure of emotional arousal (Hamm and Vaitl, 1996; Leuchs et al., 2019). In the present study, there are some inconsistencies in the measured results of FPS and SCR. No reinstatement effect could be indicated by differential SCR in all groups. A possible explanation is that the unsignaled reminder shocks after re-extinction caused a generalization of the previously acquired fear of the control stimulus, which has been shown in previous studies (Sevenster et al., 2012; Junjiao et al., 2019). SCR is a more general arousal measure, so it is more sensitive to this change than FPS, leading to an increased SCR response to CS-. The outcome, however, was further decreasing the differential SCR which distinguished responses to CS+ and CS–. This is consistent with the study by Soeter and Kindt (2011). The results of spontaneous recovery on FPS and SCR measurements are consistent because no unsignaled reminder shocks were presented before re-extinction.

In our study, the difference in memory strength caused by unpredictably timed shocks could not be detected in the acquisition. This difference did not appear until the reactivation sessions, consistent with a previous study that manipulated fear strength in humans (Kitamura et al., 2020). Kitamura et al. (2020) manipulated memory strength by changing the rate of learned reinforcement. They found that the SCR difference caused by low and high partial reinforcement was reflected during memory retrieval, and this difference significantly predicted recovery of fear at test. Even in the animal study by Amadi et al. (2017), the percentage of time that the rats in both predictable-shock and unpredictable-shock conditions exhibited freezing behavior during the acquisition was statistically indistinguishable. The group difference was found only in the interval after the second pairing of tone and foot shock and in the following recall test. Our analysis did not focus on the fear responses between the stimulus interval due to the differences between human and animal research paradigms. In future human studies, it may be possible to directly detect the enhanced fear from the stimulus presentation interval in the acquisition by improving the measurement method.

The setting of PE in the studies by Sevenster et al. (2013, 2014) on human-conditioned fear memory provided important insights into the experimental manipulation of prediction error and inspired a series of studies in this area. However, a few points of the protocol itself are open to debate. One of these is that a 50% interval reinforcement schedule cannot guarantee that participants form an expectation of the CS presence being followed by shocks every other trial. Another potential drawback is that this manner of inducing PE includes different numbers of CS presentation on different conditions during retrieval, which probably brings in irrelevant variables such as retrieval strength. Therefore, we explored an improved paradigm of PE setting, where participants could expect each CS to be followed by two shocks (US) through a 100% reinforcement schedule of fear conditioning and a CS presented during retrieval only followed by one shock. This manipulation is comparable to the US alone retrieval (Liu et al., 2014) and the over-expectation procedure (separate acquisition and compound retrieval with the equal reinforcement) (Reichelt and Lee, 2013) which induces the reconsolidation process by PE because it is over-expected. We believe that a 100% reinforcement schedule can form an explicit expectation in order to control individual differences. Furthermore, the number of retrieval trials was equal between the PE and no PE retrieval conditions, avoiding the possible effect of retrieval duration.

Based on the present settings, we tested two approaches to increasing the degree of PE in experiment 2: one is to increase the number of retrieval trials to generate multiple PE, the other is to enlarge the mismatch between what was acquired the day before and what actually appeared in the retrieval. Presenting an isolated, unreinforced CS trial is a common retrieval method for the retrieval-extinction procedure. Numerous studies have shown that a single unreinforced CS given shortly before extinction reduces spontaneous recovery and reinstatement of fear (Lee et al., 2017; Monfils and Holmes, 2018). In this study, there was no spontaneous recovery but a significant reinstatement effect in the unreinforced CS retrieval group, probably because of the enhanced fear memory acquired. It is difficult to determine from this result whether an unreinforced CS retrieval destabilizes the original memory trace or merely enhances the extinction memory caused by subsequent extinction training. However, since no significant spontaneous recovery and reinstatement exists in the multiple PE retrieval group, we can infer that multiple PE retrieval can destabilize the enhanced fear memory. Although results showed that the multiple PEs retrieval group prevented recovery more successfully than the unreinforced CS retrieval group, we cannot infer which approach produces more degree of PE. Hence, further studies are needed to keep on exploring the experimental model to build different degree of PE.

Although prediction error has been documented to be a pivotal condition to destabilize memory, the conclusion was primarily based on pharmacological intervention studies (Beckers and Kindt, 2017). Recently, other workers have argued that behavioral updating during destabilization does not require PE (Monfils and Holmes, 2018). Thus, consensus has not been attained yet on whether or not the behavioral intervention of fear memory destabilization still needs prediction error. However, our results indicate that PE is critical and necessary to trigger destabilization in retrieval-extinction, congruent with previous studies (Chen et al., 2018; Das et al., 2018; Sinclair and Barense, 2018; Junjiao et al., 2019). The aim of reconsolidation constitutes to update previously consolidated memories with new data to enhance environmental adaptation (Lee, 2009). In fear-conditioning studies, novel information consists of two sources: (1) the novelty of CS-US association violates expectancy of presentation or timing of US (Rescorla and Wagner, 1972; Fernandez et al., 2016b) and (2) the novelty of CS itself such as reminder duration and reduplication mediates the trace dominance of original and extinction memory (Li et al., 2017; Hu et al., 2018). A literature review by Fernandez et al. (2016b) indicates that PE can broadly be categorized into US processing models, CS processing models, and integration between models. That is, PE is a type of novel information that memory updates, regardless of the type of postretrieval interference.

Memory destabilization is the gateway to reconsolidation and assures a series of interventions based on the reconsolidation interference. Accumulating evidence from several studies (Diaz-Mataix et al., 2013; Sevenster et al., 2014; Alfei et al., 2015) suggests that the degree of PE during retrieval demarcates the shift from solo retrieval to reconsolidation, limbo, or extinction. Our current and previous studies also support this (Chen et al., 2018; Junjiao et al., 2019). These characteristics are referred to as reactivation-related factors, which constitute a type of boundary condition. The other type of boundary conditions are memory-related factors (e.g., age and strength of the memory) (Meir Drexler and Wolf, 2018; Wideman et al., 2018). A recent new viewpoint claims that the boundary conditions are not fixed but are variable due to the interplay between reactivation-related factors and memory-related factors (Fernandez et al., 2016a,b). This viewpoint is in accord with our observations that different degrees of PE are needed to destabilize enhanced fear memory and ordinary fear memory. The finding could be explained by the dynamic nature of memory: the brain decides the conditions of memory updating according to the characteristics of the memory itself. Furthermore, the formation and extinction of fear memory involve some critical brain areas. The amygdala is pivotal for threat memory generation and the engram cells in the basolateral amygdala are affected by some upstream brain areas. For example, the posterior insula delivers aversive somatosensory information to the amygdala for learning about prospective dangers in the environment (Berret et al., 2019), and the hippocampus sends error signals to promote the fear memory strength by escalating excitability or synchrony among neurons in the amygdala (Amadi et al., 2017). In addition, β-adrenergic signaling induces a labile state of fear memory by increasing hippocampal CA1 neuronal membrane excitability (Lim et al., 2018), indicating that upstream brain areas are also important for memory destabilization. Therefore, we speculate that the formation and destabilization of fear memory traces may share some common mechanisms. Hence, the boundary conditions of memory reconsolidation should be regarded as a “combined” factor, and both aspects should be considered when deciding the procedure to initiate memory destabilization.

There are several limitations to the present study. Firstly, on the analyses of reinstatement test, a statistically remarkable effect of the interplay between the trials and groups is lacking. One potential reason for this could be the limited sample size, which should be increased in future studies. Secondly, we do not use declarative US-expectancy ratings to measure PE in the current study, because we found that the online US-expectancy ratings may affect the sensitivity of SCR to conditioned fear in a pilot study. The study by Warren et al. (2014) also found that the inclusion of US-expectancy measures could affect the fear acquisition, extinction during reconsolidation, and reinstatement. However, the measurement of the degree of PE still needs an index. We will try to conduct a follow-up study using only FPS and tracking the US-expectancy across the different sessions further.

Finally, the primary limitation is that the control group used in our experimental design is the no PE retrieval extinction, not the standard (no retrieval) extinction. According to previous studies, retrieval without PE before intervention (behavioral or pharmacological) did not impede the return of fear, which is similar to the no retrieval intervention (Sevenster et al., 2013, 2014; Chen et al., 2018; Junjiao et al., 2019). In the present study, our hypothesis is based on previous evidence, that is, retrieval with PE can destabilize original memory, while retrieval without PE cannot (equivalent to no retrieval). However, if the no retrieval group is added as the control group, it can be more confirmed that the reconsolidation process is at work. In order to achieve more precise and convincing evidence, a no retrieval group should be included. Because of the absence of the no retrieval group, current results are only a preliminary exploration of the interaction between the two boundary conditions of memory strength and PE. We will make a stricter experimental design in our future explorations.

In conclusion, the present study suggests that boundary conditions of reconsolidation could be overcome through affecting the interplay between memory- and reactivation-related factors. These findings have remarkable implications for the clinical translation of emotional memory reconsolidation interferences. For clinical patients who are different in onset of symptoms, strength, and other characteristics, it is not feasible to use the same treatment parameters to intervene. To maximize the effect of memory reconsolidation intervention, it is imperative to select an appropriate memory retrieval scheme according to the actual situation of the patient, the critical factor of which is to produce an appropriate degree of PE.

## Data Availability Statement

The raw data supporting the conclusions of this article will be made available by the authors, without undue reservation.

## Ethics Statement

The studies involving human participants were reviewed and approved by human research ethics committee for non-clinical faculties of South China Normal University. The patients/participants provided their written informed consent to participate in this study.

## Author Contributions

XZ and WC designed the experiment and worked on the final version of the manuscript. WC, JL, LX, SZ, and MF collected and analyzed the data. All authors contributed to the article and approved the submitted version.

## Conflict of Interest

The authors declare that the research was conducted in the absence of any commercial or financial relationships that could be construed as a potential conflict of interest.

## References

[B1] Agren T. Bjorkstrand J. Fredrikson M. (2017). Disruption of human fear reconsolidation using imaginal and *in vivo* extinction. Behav. Brain Res. 319, 9–15. 10.1016/j.bbr.2016.11.014 27840245

[B2] Alfei J. M. Ferrer Monti R. I. Molina V. A. Bueno A. M. Urcelay G. P. (2015). Prediction error and trace dominance determine the fate of fear memories after post-training manipulations. Learn. Mem. 22, 385–400. 10.1101/lm.038513.115 26179232 PMC4509917

[B3] Amadi U. Lim S. H. Liu E. Baratta M. V. Goosens K. A. (2017). Hippocampal processing of ambiguity enhances fear memory. Psychol. Sci. 28, 143–161. 10.1177/0956797616674055 28182526 PMC5308550

[B4] Beck A. T. Ward C. H. Mendelson M. Mock J. Erbaugh J. (1961). An inventory for measuring depression. Arch. Gen. Psychiatry 4, 561–571. 10.1001/archpsyc.1961.01710120031004 13688369

[B5] Beckers T. Kindt M. (2017). Memory reconsolidation interference as an emerging treatment for emotional disorders: strengths, limitations, challenges, and opportunities. Annu. Rev. Clin. Psychol. 13, 99–121. 10.1146/annurev-clinpsy-032816-045209 28375725 PMC5424072

[B6] Berret E. Kintscher M. Palchaudhuri S. Tang W. Osypenko D. Kochubey O.. (2019). Insular cortex processes aversive somatosensory information and is crucial for threat learning. Science 364:eaaw0474. 10.1126/science.aaw0474 31097492

[B7] Bjorkstrand J. Agren T. Ahs F. Frick A. Larsson E. M. Hjorth O.. (2016). Disrupting reconsolidation attenuates long-term fear memory in the human amygdala and facilitates approach behavior. Curr. Biol. 26, 2690–2695. 10.1016/j.cub.2016.08.022 27568591

[B8] Blumenthal T. D. Cuthbert B. N. Filion D. L. Hackley S. Lipp O. V. van Boxtel A. (2005). Committee report: guidelines for human startle eyeblink electromyographic studies. Psychophysiology 42, 1–15. 10.1111/j.1469-8986.2005.00271.x 15720576

[B9] Bos M. G. Schuijer J. Lodestijn F. Beckers T. Kindt M. (2014). Stress enhances reconsolidation of declarative memory. Psychoneuroendocrinology 46, 102–113. 10.1016/j.psyneuen.2014.04.011 24882163

[B10] Chen W. Li J. Caoyang J. Yang Y. Hu Y. Zheng X. (2018). Effects of prediction error on post-retrieval extinction of fear to compound stimuli. Acta Psychologica Sinica 50, 739–749. 10.3724/SP.J.1041.2018.00739

[B11] Chen W. Li J. Ling X. Zhang X. Zheng X. (2020). Behavioral intervention of emotional memory reconsolidation: from bench to bedside. Adv. Psychol. Sci. 28, 240–251. 10.3724/SP.J.1042.2020.0024029553773

[B12] Chen Y. Y. Zhang L. B. Li Y. Meng S. Q. Gong Y. M. Lu L.. (2019). Post-retrieval extinction prevents reconsolidation of methamphetamine memory traces and subsequent reinstatement of methamphetamine seeking. Front. Mol. Neurosc., 12:157. 10.3389/fnmol.2019.00157 31312119 PMC6614190

[B13] Christopoulos G. I. Uy M. A. Yap W. J. (2016). The body and the brain: measuring skin conductance responses to understand the emotional experience. Organ. Res. Methods, 22, 394–420. 10.1177/1094428116681073

[B14] Das R. K. Gale G. Hennessy V. Kamboj S. K. (2018). A prediction error-driven retrieval procedure for destabilizing and rewriting maladaptive reward memories in hazardous drinkers. J. Vis. Exp. 131:e56097. 10.3791/56097 29364255 PMC5908434

[B15] Debiec J. Ledoux J. E. (2004). Disruption of reconsolidation but not consolidation of auditory fear conditioning by noradrenergic blockade in the amygdala. Neuroscience 129, 267–272. 10.1016/j.neuroscience.2004.08.018 15501585

[B16] Diaz-Mataix L. Ruiz Martinez R. C. Schafe G. E. LeDoux J. E. Doyere V. (2013). Detection of a temporal error triggers reconsolidation of amygdala-dependent memories. Curr. Biol. 23, 467–472. 10.1016/j.cub.2013.01.053 23453952 PMC3606686

[B17] Faliagkas L. Rao-Ruiz P. Kindt M. (2018). Emotional memory expression is misleading: delineating transitions between memory processes. Curr. Opin. Behav. Sci. 19, 116–122. 10.1016/j.cobeha.2017.12.018

[B18] Fernandez R. S. Bavassi L. Forcato C. Pedreira M. E. (2016a). The dynamic nature of the reconsolidation process and its boundary conditions: evidence based on human tests. Neurobiol. Learn. Mem. 130, 202–212. 10.1016/j.nlm.2016.03.001 26952269

[B19] Fernandez R. S. Boccia M. M. Pedreira M. E. (2016b). The fate of memory: reconsolidation and the case of prediction error. Neurosci. Biobehav. Rev. 68, 423–441. 10.1016/j.neubiorev.2016.06.004 27287939

[B20] Ferrer Monti R. I. Alfei J. M. Mugnaini M. Bueno A. M. Beckers T. Urcelay G. P.. (2017). A comparison of behavioral and pharmacological interventions to attenuate reactivated fear memories. Learn. Mem. 24, 369–374. 10.1101/lm.045385.117 28716956 PMC5516684

[B21] Hamm A. O. Vaitl D. (1996). Affective learning: awareness and aversion. Psychophysiology. 33, 698–710. 10.1111/j.1469-8986.1996.tb02366.x 8961792

[B22] Hu J. Wang W. Homan P. Wang P. Zheng X. Schiller D. (2018). Reminder duration determines threat memory modification in humans. Sci. Rep. 8:8848 10.1038/s41598-018-27252-0 29891856 PMC5995965

[B23] Junjiao L. Wei C. Jingwen C. Yanjian H. Yong Y. Liang X.. (2019). Role of prediction error in destabilizing fear memories in retrieval extinction and its neural mechanisms. Cortex 121, 292–307. 10.1016/j.cortex.2019.09.003 31669978

[B24] Kindt M. Soeter M. Vervliet B. (2009). Beyond extinction: erasing human fear responses and preventing the return of fear. Nat. Neurosci. 12, 256–258. 10.1038/nn.2271 19219038

[B25] Kitamura H. Johnston P. Johnson L. Strodl E. (2020). Boundary conditions of post-retrieval extinction: a direct comparison of low and high partial reinforcement. Neurobiol. Learn. Mem. 174:107285. 10.1016/j.nlm.2020.107285 32745600

[B26] Kredlow M. A. Orr S. P. Otto M. W. (2018). Exploring the boundaries of post-retrieval extinction in healthy and anxious individuals. Behav. Res. Ther. 108, 45–57. 10.1016/j.brat.2018.06.010 29981938 PMC7437718

[B27] Kredlow M. A. Unger L. D. Otto M. W. (2016). Harnessing reconsolidation to weaken fear and appetitive memories: a meta-analysis of post-retrieval extinction effects. Psychol. Bull. 142, 314–336. 10.1037/bul0000034 26689086 PMC4760909

[B28] Lee J. L. (2009). Reconsolidation: maintaining memory relevance. Trends Neurosci. 32, 413–420. 10.1016/j.tins.2009.05.002 19640595 PMC3650827

[B29] Lee J. L. C. Nader K. Schiller D. (2017). An update on memory reconsolidation updating. Trends Cogn. Sci. 21, 531–545. 10.1016/j.tics.2017.04.006 28495311 PMC5605913

[B30] Leuchs L. Schneider M. Spoormaker V. I. (2019). Measuring the conditioned response: a comparison of pupillometry, skin conductance, and startle electromyography. Psychophysiology 56:e13283. 10.1111/psyp.13283 30259985

[B31] Li J. Chen W. Caoyang J. Wu W. Jie J. Xu L.. (2017). Moderate partially reduplicated conditioned stimuli as retrieval cue can increase effect on preventing relapse of fear to compound stimuli. Front. Hum. Neurosci. 11:575. 10.3389/fnhum.2017.00575 29249946 PMC5714856

[B32] Lim C. S. Kim J. I. Kwak C. Lee J. Jang E. H. Oh J.. (2018). beta-Adrenergic signaling is required for the induction of a labile state during memory reconsolidation. Brain Res. Bull. 141, 50–57. 10.1016/j.brainresbull.2018.04.011 29680772

[B33] Liu J. Zhao L. Xue Y. Shi J. Suo L. Luo Y.. (2014). An unconditioned stimulus retrieval extinction procedure to prevent the return of fear memory. Biol. Psychiatry 76, 895–901. 10.1016/j.biopsych.2014.03.027 24813334 PMC4480632

[B34] Lonsdorf T. B. Menz M. M. Andreatta M. Fullana M. A. Golkar A. Haaker J.. (2017). Don't fear 'fear conditioning': methodological considerations for the design and analysis of studies on human fear acquisition, extinction, and return of fear. Neurosci. Biobehav. Rev. 77, 247–285. 10.1016/j.neubiorev.2017.02.026 28263758

[B35] Meir Drexler S. Wolf O. T. (2018). Behavioral disruption of memory reconsolidation: from bench to bedside and back again. Behav. Neurosci. 132, 13–22. 10.1037/bne0000231 29553773

[B36] Merlo E. Milton A. L. Goozee Z. Y. Theobald D. E. Everitt B. J. (2014). Reconsolidation and extinction are dissociable and mutually exclusive processes: behavioral and molecular evidence. J. Neurosci. 34, 2422–2431. 10.1523/JNEUROSCI.4001-13.2014 24523532 PMC3921417

[B37] Misanin J. R. Miller R. R. Lewis D. J. (1968). Retrograde amnesia produced by electroconvulsive shock after reactivation of a consolidated memory trace. Science 160, 554–555. 10.1126/science.160.3827.554 5689415

[B38] Monfils M. H. Cowansage K. K. Klann E. LeDoux J. E. (2009). Extinction-reconsolidation boundaries: key to persistent attenuation of fear memories. Science 324, 951–955. 10.1126/science.1167975 19342552 PMC3625935

[B39] Monfils M. H. Holmes E. A. (2018). Memory boundaries: opening a window inspired by reconsolidation to treat anxiety, trauma-related, and addiction disorders. Lancet Psychiatry 5, 1032–1042. 10.1016/S2215-0366(18)30270-0 30385214

[B40] Nader K. Schafe G. E. Le Doux J. E. (2000). Fear memories require protein synthesis in the amygdala for reconsolidation after retrieval. Nature 406, 722–726. 10.1038/35021052 10963596

[B41] Norrholm S. D. Jovanovic T. Vervliet B. Myers K. M. Davis M. Rothbaum B. O.. (2006). Conditioned fear extinction and reinstatement in a human fear-potentiated startle paradigm. Learn. Mem. 13, 681–685. 10.1101/lm.393906 17142300 PMC3746591

[B42] Reichelt A. C. Lee J. L. (2013). Over-expectation generated in a complex appetitive goal-tracking task is capable of inducing memory reconsolidation. Psychopharmacology (Berl.) 226, 649–658. 10.1007/s00213-012-2934-3 23239132

[B43] Rescorla R. A. Wagner A. R. (1972). A theory of Pavlovian conditioning: variations in the effectiveness of reinforcement and nonreinforcement, in Classical Conditioning II: Current Research and Theory, ed Prokasy A. H. B. W. F. (New York, NY: Appleton-Century-Crofts), 64–99.

[B44] Schiller D. Monfils M. H. Raio C. M. Johnson D. C. Ledoux J. E. Phelps E. A. (2010). Preventing the return of fear in humans using reconsolidation update mechanisms. Nature 463, 49–53. 10.1038/nature08637 20010606 PMC3640262

[B45] Sevenster D. Beckers T. Kindt M. (2012). Retrieval per se is not sufficient to trigger reconsolidation of human fear memory. Neurobiol. Learn. Mem. 97, 338–345. 10.1016/j.nlm.2012.01.009 22406658

[B46] Sevenster D. Beckers T. Kindt M. (2013). Prediction error governs pharmacologically induced amnesia for learned fear. Science 339, 830–833. 10.1126/science.1231357 23413355

[B47] Sevenster D. Beckers T. Kindt M. (2014). Prediction error demarcates the transition from retrieval, to reconsolidation, to new learning. Learn. Mem. 21, 580–584. 10.1101/lm.035493.114 25320349 PMC4201815

[B49] Sinclair A. H. Barense M. D. (2018). Surprise and destabilize: prediction error influences episodic memory reconsolidation. Learn Mem. 25, 369–381. 10.1101/lm.046912.117 30012882 PMC6049395

[B48] Sinclair A. H. Barense M. D. (2019). Prediction error and memory reactivation: how incomplete reminders drive reconsolidation. Trends Neurosci. 42, 727–739. 10.1016/j.tins.2019.08.007 31506189

[B50] Soeter M. Kindt M. (2011). Disrupting reconsolidation: pharmacological and behavioral manipulations. Learn. Mem. 18, 357–366. 10.1101/lm.2148511 21576515

[B51] Spielberger C. D. Gorsuch R. L. Lusthene R. E. (1970). Manual for the State-Trait Anxiety Inventory. Palo Alto, CA: Consulting Psychologists Press.

[B52] Suzuki A. Josselyn S. A. Frankland P. W. Masushige S. Silva A. J. Kida S. (2004). Memory reconsolidation and extinction have distinct temporal and biochemical signatures. J. Neurosci. 24, 4787–4795. 10.1523/JNEUROSCI.5491-03.2004 15152039 PMC6729467

[B53] Thompson A. Lipp O. V. (2017). Extinction during reconsolidation eliminates recovery of fear conditioned to fear-irrelevant and fear-relevant stimuli. Behav. Res. Ther. 92, 1–10. 10.1016/j.brat.2017.01.017 28171767

[B54] van Dis E. A. M. Hagenaars M. A. Bockting C. L. H. Engelhard I. M. (2019). Reducing negative stimulus valence does not attenuate the return of fear: Two counterconditioning experiments. Behav. Res. Ther. 120:103416. 10.1016/j.brat.2019.103416 31254717

[B55] Visser R. M. Lau-Zhu A. Henson R. N. Holmes E. A. (2018). Multiple memory systems, multiple time points: how science can inform treatment to control the expression of unwanted emotional memories. Philos. Trans. R. Soc. Lond. B Biol. Sci. 373:20170209. 10.1098/rstb.2017.0209 29352036 PMC5790835

[B56] Warren V. T. Anderson K. M. Kwon C. Bosshardt L. Jovanovic T. Bradley B.. (2014). Human fear extinction and return of fear using reconsolidation update mechanisms: the contribution of on-line expectancy ratings. Neurobiol. Learn. Mem. 113, 165–173. 10.1016/j.nlm.2013.10.014 24183839 PMC4351258

[B57] Wideman C. E. Jardine K. H. Winters B. D. (2018). Involvement of classical neurotransmitter systems in memory reconsolidation: Focus on destabilization. Neurobiol. Learn. Mem. 156, 68–79. 10.1016/j.nlm.2018.11.001 30395938

[B58] Winters B. D. Tucci M. C. DaCosta-Furtado M. (2009). Older and stronger object memories are selectively destabilized by reactivation in the presence of new information. Learn. Mem. 16, 545–553. 10.1101/lm.1509909 19713353

[B59] Yang Y. Jie J. Li J. Chen W. Zheng X. (2019). A novel method to trigger the reconsolidation of fear memory. Behav. Res. Ther. 122:103461. 10.1016/j.brat.2019.103461 31585344

[B60] Zimmermann J. Bach D. R. (2020). Impact of a reminder/extinction procedure on threat-conditioned pupil size and skin conductance responses. Learn. Mem. 27, 164–172. 10.1101/lm.050211.119 32179658 PMC7079572

[B61] Zuccolo P. F. Hunziker M. H. L. (2019). A review of boundary conditions and variables involved in the prevention of return of fear after post-retrieval extinction. Behav. Process. 162, 39–54. 10.1016/j.beproc.2019.01.011 30708059

