# Supplementary material for: Destabilizing Different Strengths of Fear Memories Requires Different Degrees of Prediction Error During Retrieval
Source: Front Behav Neurosci. 2021 Jan 8;14:598924. doi: 10.3389/fnbeh.2020.598924 (PMC7820768; doi:10.3389/fnbeh.2020.598924)
Supplement: Supplementary file 1 [file Table_1.DOCX]

Supplementary Material

# Supplementary Figures and Tables

## Supplementary Figures


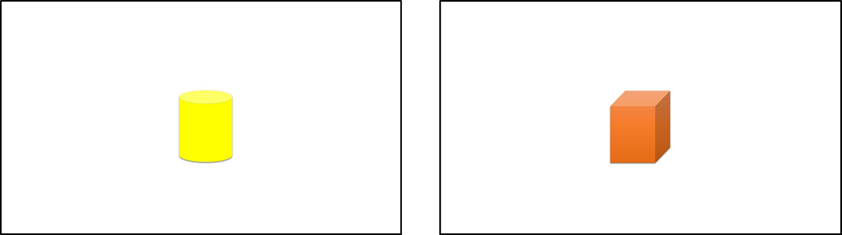


**Supplementary Figure 1.** CSs of the fear conditioning procedure. Assignment of the slides as CS1 and CS2 was counterbalanced across participants.

## Supplementary Tables

**Supplementary Table 1.** Mean values (SD) of age, trait anxiety (STAI-T) and shock intensity for the experimental groups.

|  | Experiment 1 | | |  | Experiment 2 | |
| --- | --- | --- | --- | --- | --- | --- |
| Group | predictable-shock  /PE-retrieval | predictable-shock  /no PE-retrieval | unpredictable-shock  /PE-retrieval |  | multiple PEs retrieval | unreinforced CS retrieval |
| Group size | n=18, 5 men | n=19, 5 men | n=16, 5 men |  | n=17, 5 men | n=17, 6 men |
| Age (years) | 21.7 (2.8) | 20.8 (2.1) | 20.9 (1.6) |  | 19.1 (1.1) | 19.2 (0.7) |
| Trait Anxiety | 40.2 (9.3) | 41.9 (8.5) | 36.9 (11.3) |  | 40.1 (7.2) | 39.8 (8.2) |
| Shock Intensity (V) | 49.8 (11.9) | 44 (11.3) | 42 (9.3) |  | 47.9 (9.5) | 50.1 (10.8) |
